# Correlates of Neutralization against SARS-CoV-2 Variants of Concern by Early Pandemic Sera

**DOI:** 10.1128/JVI.00404-21

**Published:** 2021-06-24

**Authors:** Samuel J. Vidal, Ai-ris Y. Collier, Jingyou Yu, Katherine McMahan, Lisa H. Tostanoski, John D. Ventura, Malika Aid, Lauren Peter, Catherine Jacob-Dolan, Tochi Anioke, Aiquan Chang, Huahua Wan, Ricardo Aguayo, Debby Ngo, Robert E. Gerszten, Michael S. Seaman, Dan H. Barouch

**Affiliations:** a Center for Virology and Vaccine Research, Beth Israel Deaconess Medical Center, Harvard Medical School, Boston, Massachusetts, USA; b Division of Infectious Diseases, Massachusetts General Hospital and Brigham and Women’s Hospital, Harvard Medical School, Boston, Massachusetts, USA; c Department of Obstetrics and Gynecology, Beth Israel Deaconess Medical Center, Harvard Medical School, Boston, Massachusetts, USA; d Harvard Medical School, Boston, Massachusetts, USA; e Program in Immunology, Harvard Medical School, Boston, Massachusetts, USA; f Division of Cardiovascular Medicine, Beth Israel Deaconess Medical Center, Harvard Medical School, Boston, Massachusetts, USA; g Ragon Institute of MGH, MIT and Harvard, Cambridge, Massachusetts, USA; h Massachusetts Consortium on Pathogen Readiness, Boston, Massachusetts, USA; University of Texas Southwestern Medical Center

**Keywords:** SARS-CoV-2, T cells, neutralizing antibodies

## Abstract

Emerging SARS-CoV-2 variants of concern that overcome natural and vaccine-induced immunity threaten to exacerbate the COVID-19 pandemic. Increasing evidence suggests that neutralizing antibody (NAb) responses are a primary mechanism of protection against infection. However, little is known about the extent and mechanisms by which natural immunity acquired during the early COVID-19 pandemic confers cross-neutralization of emerging variants. In this study, we investigated cross-neutralization of the B.1.1.7 and B.1.351 SARS-CoV-2 variants in a well-characterized cohort of early pandemic convalescent subjects. We observed modestly decreased cross-neutralization of B.1.1.7 but a substantial 4.8-fold reduction in cross-neutralization of B.1.351. Correlates of cross-neutralization included receptor binding domain (RBD) and N-terminal domain (NTD) binding antibodies, homologous NAb titers, and membrane-directed T cell responses. These data shed light on the cross-neutralization of emerging variants by early pandemic convalescent immune responses.

**IMPORTANCE** Widespread immunity to SARS-CoV-2 will be necessary to end the COVID-19 pandemic. NAb responses are a critical component of immunity that can be stimulated by natural infection as well as vaccines. However, SARS-CoV-2 variants are emerging that contain mutations in the spike gene that promote evasion from NAb responses. These variants may therefore delay control of the COVID-19 pandemic. We studied whether NAb responses from early COVID-19 convalescent patients are effective against the two SARS-CoV-2 variants, B.1.1.7 and B.1.351. We observed that the B.1.351 variant demonstrates significantly reduced susceptibility to early pandemic NAb responses. We additionally characterized virological, immunological, and clinical features that correlate with cross-neutralization. These studies increase our understanding of emerging SARS-CoV-2 variants.

## INTRODUCTION

The COVID-19 pandemic has resulted in more than 110 million infections and 2.5 million deaths worldwide (1). Accumulating evidence suggests that both natural (2, 3) and vaccine-induced (4–7) immunity strongly protects from SARS-CoV-2. However, the emergence of novel SARS-CoV-2 variants with diverse mutations threatens to attenuate the magnitude of these mechanisms of immunity (8). Adoptive transfer (9) and vaccine studies (10) in rhesus macaques suggest that neutralizing antibody (NAb) responses are a critical correlate of protection against SARS-CoV-2 infection. Therefore, the extent to and mechanisms by which early pandemic convalescent-phase sera cross-neutralize emerging variants is an important area of investigation.

The first major SARS-CoV-2 mutation to be extensively characterized was D614G, which began to emerge in April 2020. Epidemiological and virological studies suggest that D614G exhibits increased replicative capacity (11–13) while also conferring increased cross-neutralization by vaccine-induced mouse and monkey antibody responses as well as convalescent human responses (14). Subsequently, reports began to emerge in the United Kingdom in December 2020 regarding the B.1.1.7 variant that contains 8 spike mutations in addition to D614G (15). As-yet unpublished reports suggest that the N501Y mutation located in the receptor binding domain (RBD) may increase its affinity to the angiotensin-converting enzyme 2 (ACE2) receptor (16), while the Δ69-to-70 deletion in the N-terminal domain (NTD) may increase SARS-CoV-2 infectivity (17). Clinically, the B.1.1.7 variant is associated with increased viral loads (18). In addition, reports began to emerge in South Africa in December 2020 regarding the B.1.351 variant that contains 9 spike mutations in addition to D614G. These involve clusters of mutations in the RBD and NTD, including the K417N and E484K mutations that promote neutralization resistance (19–21). Other B.1.1.7 and B.1.351 spike mutations are presently of uncertain significance.

Published and as-yet unpublished reports are emerging regarding the cross-neutralizing potential of clinically relevant monoclonal antibodies and vaccine-induced responses. mRNA-1273 (21, 22) and BNT162b2 (21, 23) vaccine-induced sera showed efficient cross-neutralization of B.1.1.7. In contrast, mRNA-1273 (21, 22) and BNT162b2 (21, 24) vaccine-induced sera showed ∼3- to 8-fold reductions in cross-neutralization of B.1.351. The REGN-COV2 antibody cocktail showed a ∼10-fold reduction in cross-neutralization of B.1.351 in one unpublished report (25). In another unpublished report, REGN-COV2 retained cross-neutralization of B.1.351, while LY-CoV555 showed a >100-fold reduction in 50% inhibitory concentration (IC_50_) (21).

Additional emerging reports suggest 1- to 4-fold and 3- to 30-fold reductions in the neutralization of B.1.1.7 and B.1.351, respectively, by early COVID-19 pandemic convalescent-phase sera (21, 26, 27). However, overall, little is known about the extent and mechanisms by which natural immunity acquired during the early COVID-19 pandemic confers cross-neutralization of emerging variants. In the present study, we examined these questions in a well-characterized cohort of early COVID-19 pandemic convalescent subjects.

## RESULTS

### Early COVID-19 pandemic convalescent cohort and prepandemic controls.

We identified 21 patients admitted to Beth Israel Deaconess Medical Center (BIDMC; Boston, MA, USA) between April and June of 2020 for symptoms related to COVID-19 (Table 1). All patients had a documented positive SARS-CoV-2 nasopharyngeal nucleic acid amplification test at admission. Ages ranged from 33 to 94 (median age 68), and there were 11 females and 10 males. Comorbidity numbers ranged from 0 to 5 (median, 3). Hospital stay ranged from 2 to 34 days (median stay, 15 days). Twenty out of twenty-one patients developed severe COVID-19 by World Health Organization criteria (peripheral oxygen saturation [SpO_2_] < 94%), and among these, 10 patients required intubation. Serum and peripheral blood mononuclear cells (PBMCs) were obtained within 2 days of discharge and after resolution or near resolution of presenting symptoms. We additionally obtained serum and PBMCs from 8 archival adult prepandemic controls.

**TABLE 1:** Early pandemic convalescent COVID-19 inpatient cohort clinical data^*a*^

Patient no.	Age (yrs)	Gender	Major comorbidity(ies)	Admission duration (no. of days)	Location of peak oxygen requirement
1	70	M	Degenerative joint disease	6	Nasal cannula
2	59	F	Hypertension, diabetes mellitus, deep vein thrombosis	5	Nasal cannula
3	69	F	Obesity, hypertension, diabetes mellitus, obstructive sleep apnea, rheumatoid arthritis	34	Intubated
4	59	F	Obesity, hypertension	4	Nasal cannula
5	68	F	Pulmonary embolus, sarcoidosis on prednisone, hypothyroidism, stroke	25	Intubated
6	39	M	None	20	Intubated
7	68	M	Diabetes mellitus	32	Intubated
8	82	M	Stroke, cognitive impairment, seizure	14	Nasal cannula
9	80	F	Hypertension, breast cancer on hormonal therapy, cognitive impairment	6	Room air
10	77	M	Hypertension, diabetes mellitus, cognitive impairment	33	Intubated
11	50	F	Obesity, diabetes mellitus	25	Intubated
12	73	M	Hypertension, diabetes mellitus, cognitive impairment	12	Nasal cannula
13	81	M	Hypertension, diabetes mellitus	25	Intubated
14	90	M	Arrhythmia, chronic obstruction pulmonary disease, chronic kidney disease, cognitive impairment	15	Nasal cannula
15	66	F	Reactive airway disease	34	Intubated
16	57	F	Hypertension, diabetes mellitus, muscular dystrophy	11	Nasal cannula
17	33	F	Obesity, reactive airway disease	5	Nasal cannula
18	94	F	Hypertension, arrhythmia	2	Nasal cannula
19	84	M	Hypertension, diabetes mellitus, Parkinson disease	9	Nasal cannula
20	54	F	Obesity, hypertension, diabetes mellitus, obstructive transplant on multimodal immunosuppression	32	Intubated
21	57	M	Hypertension, diabetes mellitus, coronary artery disease, renal transplant on multimodal immunosuppression	32	Intubated

a Early pandemic convalescent COVID-19 inpatient (*n* = 21) data, including age, gender, major comorbidities, admission duration, and peak oxygen requirement.

### Characterization of an early pandemic cohort with Wuhan strain sequences.

**FIG 1:**
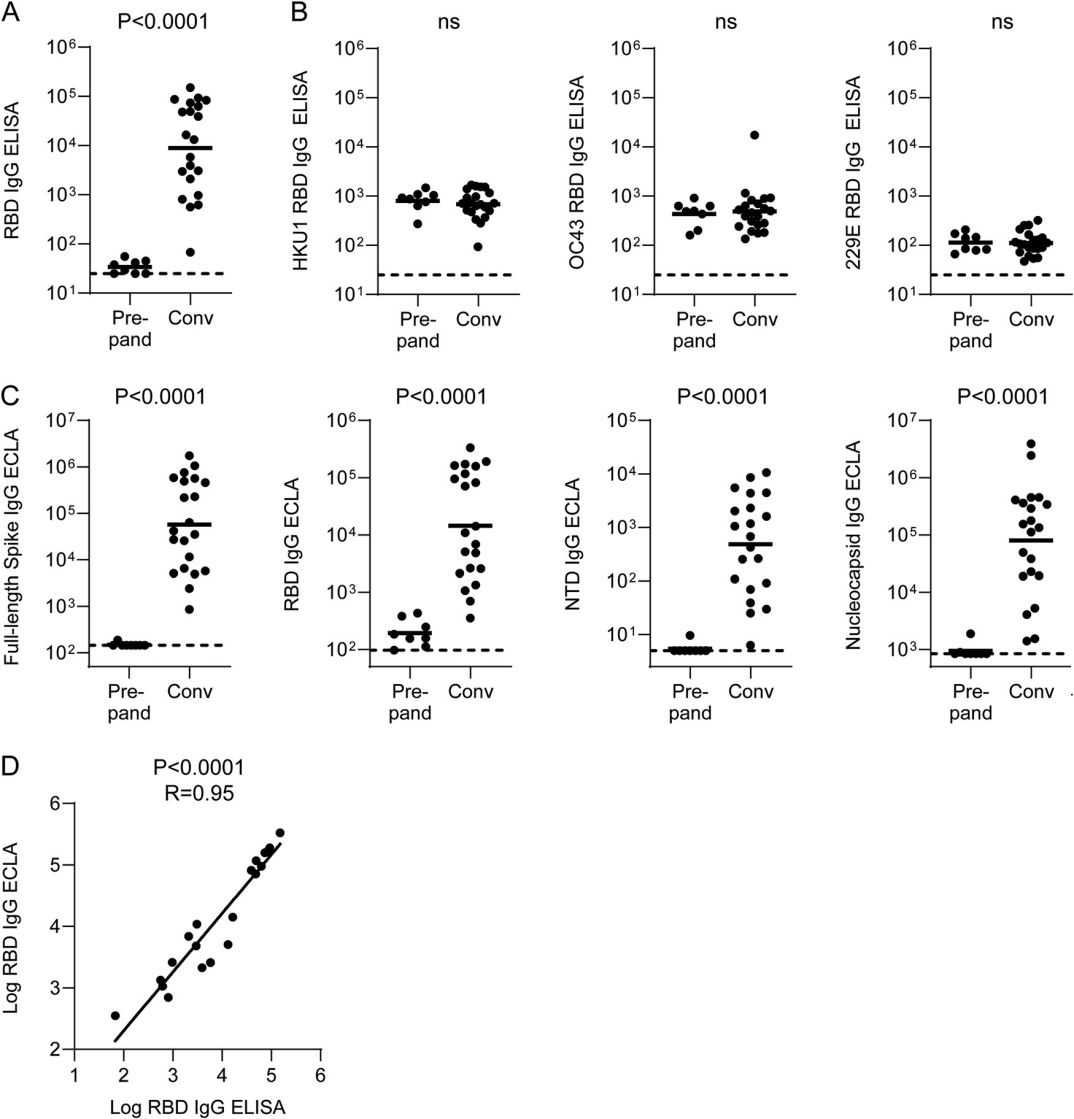
SARS-CoV-2 and endemic coronavirus binding antibody responses among early pandemic convalescent COVID-19 inpatients and prepandemic controls. (A) SARS-CoV-2 spike RBD IgG ELISA titers. (B) HKU1, OC43, and 229E spike RBD IgG ELISA titers. (C) Full-length spike, spike RBD, and spike NTD IgG ECLA signals. (D) Spearman correlation between SARS-CoV-2 spike RBD IgG ELISA titer and ECLA signal. Bars represent geometric means. Dotted lines represent limits of detection (LOD) for ELISA and lower limits of detection (LLOD) for ECLA. Convalescent COVID-19 inpatient and prepandemic subjects were compared by Mann-Whitney test.

We initially characterized our convalescent COVID-19 cohort using early pandemic (Wuhan strain) detection reagents. We first characterized binding antibody responses by enzyme-linked immunosorbent assay (ELISA) using strain-specific RBD coating antigens from SARS-CoV-2 as well as the common endemic coronaviruses HKU1, OC43, and 229E. We observed robust SARS-CoV-2 binding responses in all COVID-19 individuals except participant 14, a 90-year-old man with multiple comorbidities (Fig. 1A). In contrast, all endemic coronaviruses demonstrated similar RBD IgG ELISA titers between prepandemic and convalescent COVID-19 subjects (Fig. 1B).

In order to gain greater epitope specificity among binding antibody responses, we next used a multiplexed electrochemiluminescence assay (ECLA; Meso Scale Discovery) to measure responses to full-length SARS-CoV-2 spike, RBD, NTD, and nucleocapsid. All COVID-19 patients demonstrated reactivity greater than prepandemic controls to full-length spike (Fig. 1C). Reponses to RBD and NTD were comparable in frequency, with only participant 14 showing signal similar to prepandemic controls (Fig. 1C). Moreover, most patients also demonstrated binding antibody responses to nucleocapsid (Fig. 1C). Overall, this antibody binding epitope distribution was comparable to previous studies of convalescent individuals (28). RBD IgG responses measured by ELISA and ECLA were strongly correlated (Fig. 1D), and therefore, going forward, we used multiplexed ECLA binding data in our analyses.

**FIG 2:**
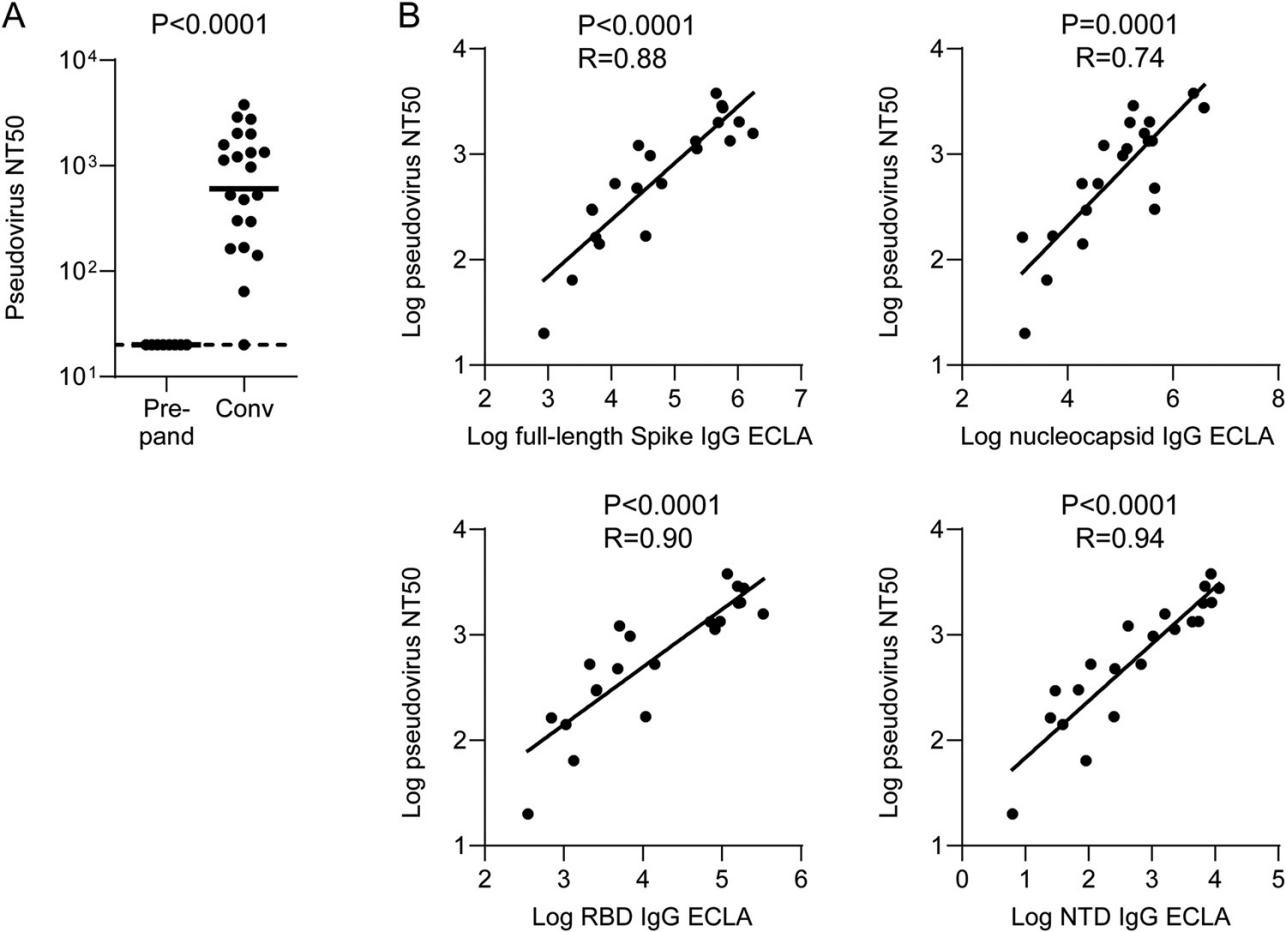
SARS-CoV2 pseudovirus neutralization among early pandemic convalescent COVID-19 inpatients and correlations with binding antibody titers. (A) Pseudovirus neutralization titers among early pandemic convalescent COVID-19 inpatients and prepandemic controls. (B) Spearman correlations between spike full-length, RBD, NTD, and nucleocapsid IgG ECLA signals and pseudovirus neutralization titers. Bars represent geometric means. Dotted lines represent LOD. Convalescent COVID-19 inpatients and prepandemic controls were compared by Mann-Whitney test.

We next assessed NAb responses in an assay employing a recombinant lentiviral pseudovirus expressing the Wuhan SARS-CoV-2 spike sequence and HEK293T target cells ectopically expressing the human ACE2 receptor (HEK293T-hACE2). We observed responses above prepandemic controls among all but one convalescent COVID-19 subjects (Fig. 2A). The frequency and magnitude of NAb responses were consistent with recent large cohort studies of convalescent individuals (29, 30). NAb responses correlated most strongly with both RBD and NTD binding antibody responses (Fig. 2B). A significant but less robust correlation between NAb responses and nucleocapsid binding antibody responses was unsurprising given that this antigen is presumably not a target for NAbs and likely serves as a marker of antibody responses (Fig. 2B).

**FIG 3:**
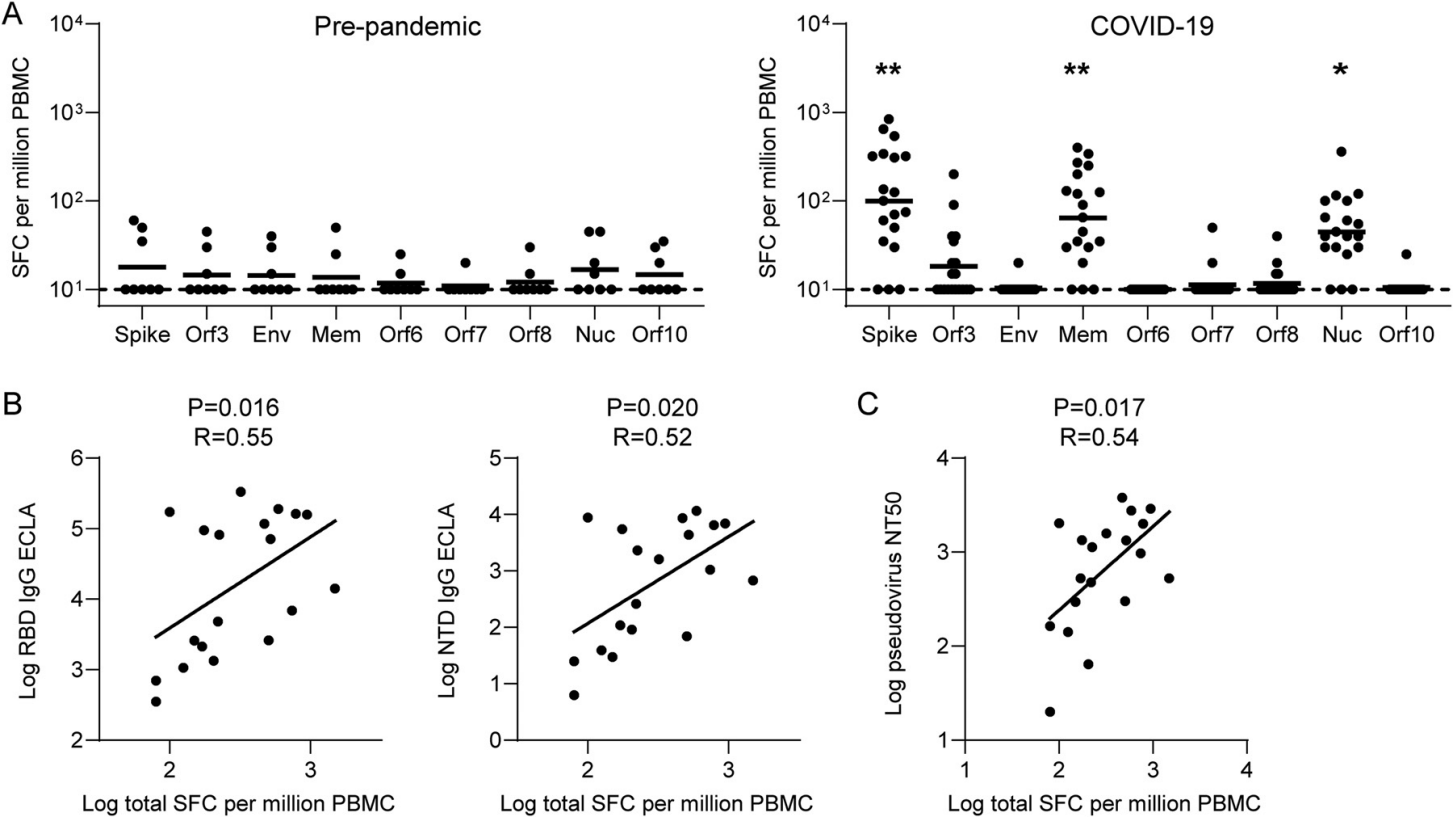
SARS-CoV-2 T cell responses and correlations with antibody responses among early pandemic convalescent COVID-19 inpatients and prepandemic controls. (A) IFN-γ ELISPOT responses to selected structural and accessory SARS-CoV-2 antigens. (B and C) Spearman correlations between aggregate IFN-γ ELISPOT responses and RBD and NTD binding (B) and pseudovirus Nab responses (C). Bars represent geometric means. Dotted lines represent LOD. Convalescent COVID-19 inpatient and prepandemic and subjects were compared by Mann-Whitney test. *, *P* < 0.05; **, *P* < 0.01.

To characterize T cell responses, we performed interferon gamma (IFN-γ) enzyme-linked immune absorbent spot (ELISPOT) assays with the convalescent COVID-19 cohort and prepandemic controls. We focused on the 9 structural and accessory proteins that account for approximately 75% of the CD4 and CD8 T cell responses to SARS-CoV-2 infection (31). We observed ELISPOT responses among all convalescent patients, primarily against the SARS-CoV-2 spike, membrane, and nucleocapsid proteins (Fig. 3A). This pattern of immunodominance was consistent with a previous report that assessed T cell responses by flow cytometry (31). Moreover, similar to prior reports (31–33), aggregate T cell responses correlated with spike RBD and NTD binding (Fig. 3B) as well as NAb (Fig. 3C) responses. We observed a trend toward correlation between aggregate T cell responses and nucleocapsid binding antibodies (*P* = 0.067, *R* = 0.43; two-sided Spearman rank correlation test).

### NAb responses to emerging SARS-CoV-2 variants in early pandemic sera.

**FIG 4:**
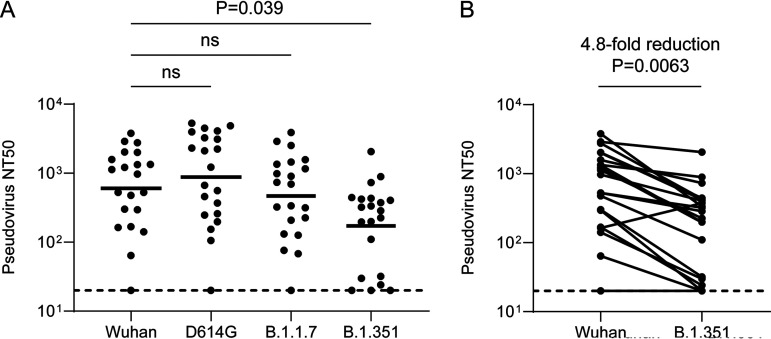
Cross-neutralizing antibody responses to emerging SARS-CoV-2 variants among early pandemic convalescent-phase sera. (A) Grouped comparison of SARS-CoV-2 Wuhan, B.1.1.7, and B.1.351 strain pseudovirus NAb titers. (B) Pairwise comparison of SARS-CoV-2 Wuhan and B.1.351 strain pseudovirus NAb titers. Bars represent geometric means. Dotted lines represent LOD. Multigroup comparisons were performed by Kruskal-Wallis test. Paired comparison was performed by Mann-Whitney test. ns, not significant.

In order to assess NAb responses to emerging SARS-CoV-2 variants by early pandemic sera, we next constructed pseudoviruses containing the B.1.1.7 and B.1.351 spike sequences for neutralization assays. We additionally constructed a pseudovirus containing only D614G since this spike variant is now ubiquitous and was previously shown to exhibit increased cross-neutralization by early pandemic convalescent-phase serum (11, 12, 14). Indeed, we observed a modest though nonsignificant increase in the neutralization geometric mean titer (GMT) of D614G among 17/20 subjects with detectable Wuhan NAb titers (average, 1.5-fold increase; range, 0.8 to 2.8; Fig. 4A).

In contrast, B.1.1.7 showed a modest though nonsignificant decrease in pseudovirus neutralization among 13/20 subjects with detectable Wuhan NAb titers (average, 1.5-fold decrease; range, 0.5 to 2.9; Fig. 4A). Notably, B.1.351 showed a significant reduction in pseudovirus neutralization among 19/20 subjects with detectable Wuhan NAb titers (average, 4.8-fold decrease; range, 0.4 to 14.8; Fig. 4A). Reductions in B.1.351 cross-neutralization were generally uniform, and the 2/20 patients with newly undetectable B.1.351 NAb titers had low baseline Wuhan titers (Fig. 4B). Notably, individuals with Wuhan NAb titers as high as 295 developed undetectable B.1.351 cross-neutralization titers.

### Correlates of emerging SARS-CoV-2 variant cross-neutralization.

**FIG 5:**
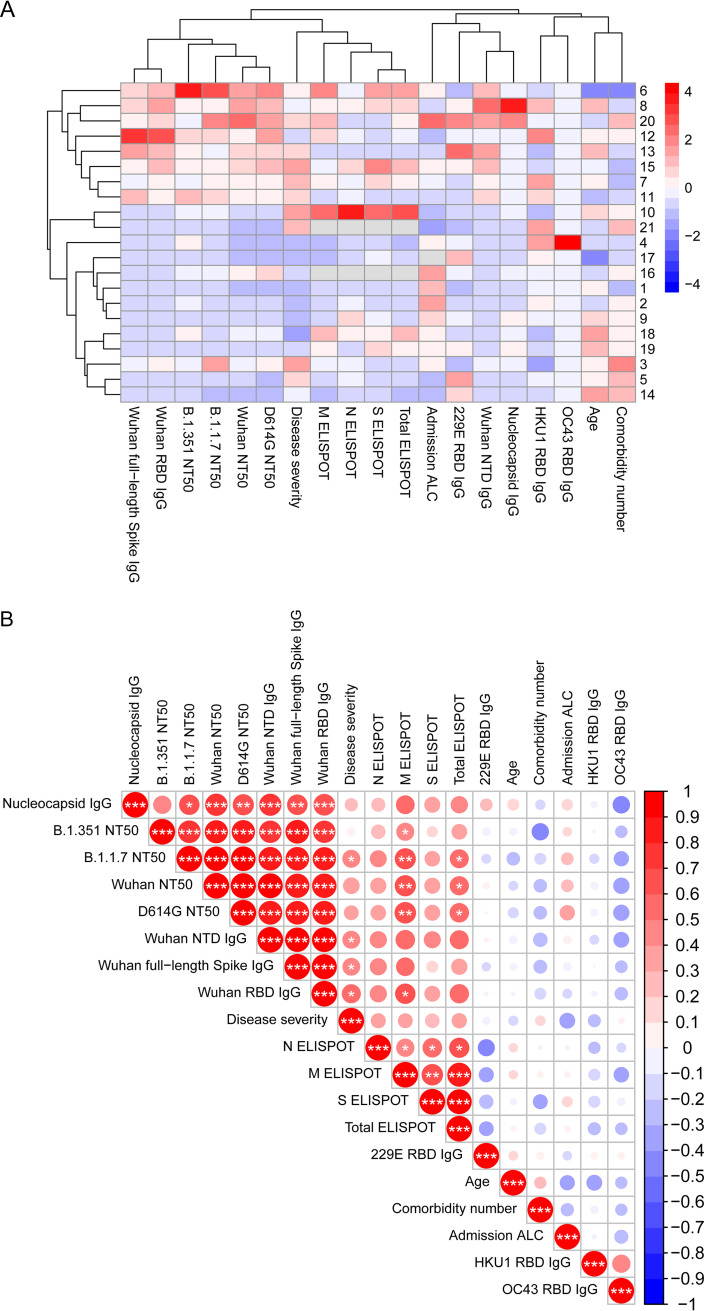
Correlates of emerging variant cross-neutralization by early pandemic convalescent-phase sera. (A) Heat map with unsupervised hierarchical clustering of clinical, virological, and immunological variables as well as patients. (B) Correlogram with unsupervised hierarchical clustering of the same clinical, virological, and immunological variables as in panel A. Red shading depicts positive correlations, blue shading depicts negative correlations, intensity of the shading represents magnitude of the Spearman *R* statistic, and size of the shading represents significance. *, *P* < 0.05; **, *P* < 0.01; ***, *P* < 0.001. All data include a Benjamini-Hochberg correction for multiple comparisons.

Given the number of clinical and immunological variables in our study, we next used unbiased bioinformatics approaches to analyze our results. We first performed unsupervised hierarchical clustering of patients and all of our continuous clinical, immunological, and virological variables (Fig. 5A). Hospital duration showed a strong association with intubation in our cohort. Average stays were 8.1 and 28.6 days among nonintubated and intubated individuals, respectively (*P* = 4.4 × 10^−9^; Student's *t* test). We therefore used this continuous variable as a surrogate for disease severity in our computational analyses. We observed that individuals with strong SARS-CoV-2-specific humoral and cellular responses formed a large cluster. Interestingly, this cluster also contained hospital duration, was associated with intubation (*P* = 0.03; Fisher’s exact test), and segregated into distinct groups of humoral or cellular immune responses (Fig. 5A).

To gain insights into the correlates of both Wuhan strain neutralization and variant cross-neutralization by early pandemic sera, we assembled a correlogram incorporating the same variables used in our heat map (Fig. 5B). Unsupervised hierarchical clustering revealed a group of highly correlated features that exclusively included binding and NAb responses to SARS-CoV-2. Other than NAb responses to Wuhan, D614G, and B.1.1.7, NAb responses to B.1.351 showed the strongest correlations with Wuhan full-length spike, RBD, and NTD IgG binding antibody responses. These data suggest that baseline Wuhan spike binding and NAb responses may be the most important correlates of B.1.351 cross-neutralization. Moreover, several of the SARS-CoV-2 antibody responses correlated with disease severity as well as with ELISPOT responses. Interestingly, among cellular responses, we observed that membrane-specific responses exhibit the strongest correlations with binding and NAb responses. Finally, we observed that antibody responses to the RBD of pre-COVID-19 endemic coronaviruses showed no correlation with SARS-CoV-2 immune responses in our study.

## DISCUSSION

Little is known about the extent and mechanisms by which natural immunity acquired during the early COVID-19 pandemic confers cross-neutralization of emerging variants. In the present study, we examined these questions in a well-characterized cohort of early COVID-19 pandemic convalescent subjects. We observed modest reductions in cross-neutralization of B.1.1.7 and significant reductions in cross-neutralization of B.1.351. Correlates of cross-neutralization included RBD and NTD binding and spike NAb responses as well as membrane glycoprotein-directed T cell responses. These data shed light on cross-neutralization of emerging variants by early pandemic immune responses.

Given the recent emergence of B.1.1.7 and B.1.351, the cross-neutralization of these variants by early pandemic sera has yet to be firmly established. An initial study (21) employed a vesicular stomatitis virus pseudovirus assay with Vero cells and a combination of nonsevere and severe COVID-19 convalescent-phase sera to report 2.7- to 3.8-fold reductions in cross-neutralization of B.1.1.7 and 11- to 33-fold reductions in cross-neutralization of B.351. A second unpublished report (26) using authentic virus with Vero-hACE2-TMPRSS2 cells and mild COVID-19 convalescent-phase sera found a 4.5-fold reduction in cross-neutralization of B.1.351. A third study using authentic virus with Vero cells and severe COVID-19 sera at various stages of convalescence reported 2.1- to 4.8-fold reductions in cross-neutralization of B.351. We used a lentiviral pseudovirus assay with HEK293T-hACE2 cells and a relatively homogeneous group of convalescent-phase sera following hospitalization for severe COVID-19 to report a 4.8-fold average reduction in cross-neutralization of B.351. Variability in the degree of cross-neutralization among recent studies and our data may be explained by differences in neutralization assays as well as clinical heterogeneity among study populations, though further studies are needed to establish consistent trends. Nonetheless, these data collectively suggest significant reductions but not complete effacement of B.1.351 neutralization by early pandemic convalescent-phase sera. Finally, mRNA-1273 (18, 19) and BNT162b2 (18, 21) vaccine-induced sera showed ∼3-8-fold reductions in cross-neutralization of B.1.351.

Our correlates analysis suggests that the most important predictor of Wuhan, as well as variant neutralization, may be high levels of both RBD and NTD baseline binding antibody responses. These findings both affirm the critical role of the RBD in NAb responses and corroborate a growing literature highlighting the importance of the NTD as a target (28, 34–36). Moreover, binding and NAb responses were associated with hospital duration and intubation, corroborating a now large literature linking disease severity with increased adaptive immune responses (28, 37–41). Consistent with previous reports, binding and NAb responses correlated with T cells responses (31–33). Interestingly, we observed that membrane-specific ELISPOT responses showed the strongest correlation with antibody responses. This might be explained by the finding that the spike- and nucleocapsid-specific T cell pools contain a significant proportion of cross-reactive T cell responses derived from prepandemic coronavirus infections (42, 43), although further studies with larger numbers of patients are necessary to clarify this phenomenon.

We reported that IgG derived from convalescent rhesus macaques previously challenged with Washington (USA-WA1) strain SARS-CoV-2 can confer protection from homologous challenge after adoptive transfer into naive monkeys (9). Using a dose escalation scheme and logistic regression analysis, we estimated that a homologous NAb titer of approximately 50 protected from infection. In addition to the 2 patients with baseline Wuhan NAb titers who showed newly undetectable B.1.351 cross-neutralization titers, an additional two subjects showed B.1.351 cross-neutralization titers below this threshold (Fig. 4B). Although our prior protection data were obtained in rhesus macaques lacking SARS-CoV-2-specific T cells and challenged with Washington strain, these data suggest that a fraction of early pandemic convalescent individuals may exhibit subprotective B.1.351 cross-neutralization titers.

In summary, we report the cross-neutralization of emerging SARS-CoV-2 variants of concern by early pandemic sera. Our data support significant reductions but not complete effacement of B.1.351 cross-neutralization, and we report that RBD and NTD binding and spike NAb responses, as well as membrane-directed T cell responses, are correlates of cross-neutralization. These data expand our understanding of emerging SARS-CoV-2 variants that threaten to exacerbate the COVID-19 pandemic.

## MATERIALS AND METHODS

### Patient enrollment.

Participants were enrolled under the Beth Israel Deaconess Medical Center (BIDMC) COVID-19 Tissue and Data Repository (IRB 2020P00361) between April and June 2020. Eligible participants were 18 years or older and able to provide informed consent or have a legal authorized representative consent on their behalf. All electronic health records (EHRs) were reviewed by an infectious diseases physician. Comorbidities and admission absolute lymphocyte counts (ALCs) were determined on the basis of admitting physician notes, and all other reported clinical details were determined from the EHR as appropriate. We identified eight archival prepandemic adult subjects from unrelated studies in our laboratory as controls.

### Enzyme-linked immunosorbent assay.

ELISA plates (Thermo Fisher) were coated with spike receptor binding domain (RBD) protein and stored overnight at 4°C. All ELISA proteins were synthesized at the Ragon Institute of Massachusetts General Hospital (MGH), Massachusetts Institute of Technology (MIT), and Harvard. Plates were then blocked, and serially diluted serum samples were added to each well. Following sample incubation, plates were washed with Dulbecco’s phosphate-buffered saline (DPBS)-Tween, and horseradish peroxidase (HRP)-conjugated goat anti-human immunoglobulin secondary antibodies of the indicated isotype specificity were added (IgG [Invitrogen], IgA [Bethyl], and IgM [Invitrogen]). Plates were washed, developed with KPL TMB peroxidase, the reaction was halted with KPL TMB stop solution, and absorbance was recorded using a VersaMax microplate reader.

### Multiplexed electrochemiluminescence assay.

ECLA plates (Meso Scale Discovery; SARS-CoV-2 panel 2 [IgG] kit) were incubated with blocking buffer for 30 min followed by serum diluted at 1:25,000 and 1:125,000 for 2 h. Plates were then washed and incubated with secondary antibody (Sulfo-Tag anti-human IgG antibody) for 1 h. Plates were washed again and incubated in read buffer, and luminescence was detected on a Meso Sector S 600 microplate reader. Plates were run with manufacturer-provided serially diluted stock controls. Serum dilution was selected when luminescence data were contained with the linear range of the standard curve, which was used to calculate arbitrary units per milliliter. Lower limits of detection were calculated by fitting 2.5 standard deviations above the zero calibrator to the standard curve for each individual plate.

### Pseudovirus neutralization assay.

A lentiviral pseudovirus expressing a luciferase reporter gene was generated as previously described (10). Briefly, packaging plasmid psPAX2 (AIDS Resource and Reagent Program), luciferase reporter plasmid pLenti-CMV Puro-Luc (Addgene), and spike protein expressing pcDNA3.1-SARS CoV-2 SΔCT sequences for the Wuhan, D614G, B.1.1.7, and B.1.351 strains were cotransfected into HEK293T cells by Lipofectamine 2000 (Thermo Fisher). Supernatant was collected 48 h posttransfection. HEK293T-hACE2 cells were seeded in 96-well plates at a density of 1.75 × 10^4^ cells per well overnight. Threefold serial dilutions of heat-inactivated serum samples were prepared and mixed with 50 μl of pseudovirus. The mixture was incubated at 37°C for 1 h before adding to HEK293T-hACE2 cells. Forty-eight hours after infection, cells were lysed in Steady-Glo luciferase assay (Promega). SARS-CoV-2 neutralization titers were defined as the sample dilution at which a 50% reduction in relative light unit was observed relative to the average of the virus control wells.

### IFN-γ enzyme-linked immune absorbent spot assay.

White membrane plates (Millipore) were coated at 4°C overnight with 10 μg/ml anti-human IFN-γ (Mabtech). Peptides spanning the entire nonreplicase SARS-CoV-2 genome were synthesized as 15 mers overlapping by 11 residues, and peptides were organized into individual antigen pools for PBMC stimulation (JPT Peptide Technologies). Next, rested PBMCs were plated in duplicate at 2 × 10^5^ with peptide pools corresponding to individual SARS-CoV-2 proteins at 2 μg/ml for 18 h at 37°C. Development was achieved by addition of biotin (Mabtech), anti-biotin (Vector Labs), and chromogen (Pierce). Background subtraction was performed using a matched 0.4% dimethyl sulfoxide (DMSO) control well.

### Statistical methods.

Pairwise comparisons of ELISA, ECLA, 50% neutralizing titer (NT_50_), and ELISPOT clinical data were performed by Mann-Whitney test. Multigroup comparisons were performed by Kruskal-Wallis test. These tests, as well as pairwise Spearman correlations, were performed using GraphPad Prism 8 software. The heatmap was generated using the pheatmap package in R, scaled by variable values, and clustered both by patient and by variable using default scaling and hierarchical clustering settings. For the correlogram, pairwise Spearman rank coefficients were calculated using the psych package v2.0.12 in R using the corr.test function with default settings and the adjust argument set to “fdr” to calculate adjusted *P* values using Benjamini-Hochberg correction for multiple comparisons. The resulting correlation matrix was visualized as a correlogram using the corrplot package in R. Spearman rank coefficients were ordered via hierarchical clustering by setting the order argument to “hclust” in the corrplot function.

## References

[1] Dong E, Du H, Gardner L. 2020. An interactive web-based dashboard to track COVID-19 in real time. Lancet Infect Dis 20:533–534. 10.1016/S1473-3099(20)30120-1. 32087114 PMC7159018

[2] Lumley SF, O'Donnell D, Stoesser NE, Matthews PC, Howarth A, Hatch SB, Marsden BD, Cox S, James T, Warren F, Peck LJ, Ritter TG, de Toledo Z, Warren L, Axten D, Cornall RJ, Jones EY, Stuart DI, Screaton G, Ebner D, Hoosdally S, Chand M, Crook DW, O'Donnell AM, Conlon CP, Pouwels KB, Walker AS, Peto TEA, Hopkins S, Walker TM, Jeffery K, Eyre DW, Oxford University Hospitals Staff Testing Group. 2021. Antibody status and incidence of SARS-CoV-2 infection in health care workers. N Engl J Med 384:533–540. 10.1056/NEJMoa2034545. 33369366 PMC7781098

[3] Harvey RA, Rassen JA, Kabelac CA, Turenne W, Leonard S, Klesh R, Meyer WA, III, Kaufman HW, Anderson S, Cohen O, Petkov VI, Cronin KA, Van Dyke AL, Lowy DR, Sharpless NE, Penberthy LT. 2021. Association of SARS-CoV-2 seropositive antibody test with risk of future infection. JAMA Intern Med 10.1001/jamainternmed.2021.0366. PMC7905701 33625463

[4] Polack FP, Thomas SJ, Kitchin N, Absalon J, Gurtman A, Lockhart S, Perez JL, Perez Marc G, Moreira ED, Zerbini C, Bailey R, Swanson KA, Roychoudhury S, Koury K, Li P, Kalina WV, Cooper D, Frenck RW, Jr., Hammitt LL, Tureci O, Nell H, Schaefer A, Unal S, Tresnan DB, Mather S, Dormitzer PR, Sahin U, Jansen KU, Gruber WC, Group CCT, C4591001 Clinical Trial Group. 2020. Safety and efficacy of the BNT162b2 mRNA Covid-19 vaccine. N Engl J Med 383:2603–2615. 10.1056/NEJMoa2034577. 33301246 PMC7745181

[5] Baden LR, El Sahly HM, Essink B, Kotloff K, Frey S, Novak R, Diemert D, Spector SA, Rouphael N, Creech CB, McGettigan J, Khetan S, Segall N, Solis J, Brosz A, Fierro C, Schwartz H, Neuzil K, Corey L, Gilbert P, Janes H, Follmann D, Marovich M, Mascola J, Polakowski L, Ledgerwood J, Graham BS, Bennett H, Pajon R, Knightly C, Leav B, Deng W, Zhou H, Han S, Ivarsson M, Miller J, Zaks T, COVE Study Group. 2021. Efficacy and safety of the mRNA-1273 SARS-CoV-2 vaccine. N Engl J Med 384:403–416. 10.1056/NEJMoa2035389. 33378609 PMC7787219

[6] Voysey M, Clemens SAC, Madhi SA, Weckx LY, Folegatti PM, Aley PK, Angus B, Baillie VL, Barnabas SL, Bhorat QE, Bibi S, Briner C, Cicconi P, Collins AM, Colin-Jones R, Cutland CL, Darton TC, Dheda K, Duncan CJA, Emary KRW, Ewer KJ, Fairlie L, Faust SN, Feng S, Ferreira DM, Finn A, Goodman AL, Green CM, Green CA, Heath PT, Hill C, Hill H, Hirsch I, Hodgson SHC, Izu A, Jackson S, Jenkin D, Joe CCD, Kerridge S, Koen A, Kwatra G, Lazarus R, Lawrie AM, Lelliott A, Libri V, Lillie PJ, Mallory R, Mendes AVA, Milan EP, Minassian AM, Oxford COVID Vaccine Trial Group, et al. 2021. Safety and efficacy of the ChAdOx1 nCoV-19 vaccine (AZD1222) against SARS-CoV-2: an interim analysis of four randomised controlled trials in Brazil, South Africa, and the UK. Lancet 397:99–111. 10.1016/S0140-6736(20)32661-1. 33306989 PMC7723445

[7] Logunov DY, Dolzhikova IV, Shcheblyakov DV, Tukhvatulin AI, Zubkova OV, Dzharullaeva AS, Kovyrshina AV, Lubenets NL, Grousova DM, Erokhova AS, Botikov AG, Izhaeva FM, Popova O, Ozharovskaya TA, Esmagambetov IB, Favorskaya IA, Zrelkin DI, Voronina DV, Shcherbinin DN, Semikhin AS, Simakova YV, Tokarskaya EA, Egorova DA, Shmarov MM, Nikitenko NA, Gushchin VA, Smolyarchuk EA, Zyryanov SK, Borisevich SV, Naroditsky BS, Gintsburg AL, Gam-COVID-Vac Vaccine Trial Group. 2021. Safety and efficacy of an rAd26 and rAd5 vector-based heterologous prime-boost COVID-19 vaccine: an interim analysis of a randomised controlled phase 3 trial in Russia. Lancet 397:671–681. 10.1016/S0140-6736(21)00234-8. 33545094 PMC7852454

[8] Fontanet A, Autran B, Lina B, Kieny MP, Karim SSA, Sridhar D. 2021. SARS-CoV-2 variants and ending the COVID-19 pandemic. Lancet 397:952–954. 10.1016/S0140-6736(21)00370-6. 33581803 PMC7906631

[9] McMahan K, Yu J, Mercado NB, Loos C, Tostanoski LH, Chandrashekar A, Liu J, Peter L, Atyeo C, Zhu A, Bondzie EA, Dagotto G, Gebre MS, Jacob-Dolan C, Li Z, Nampanya F, Patel S, Pessaint L, Van Ry A, Blade K, Yalley-Ogunro J, Cabus M, Brown R, Cook A, Teow E, Andersen H, Lewis MG, Lauffenburger DA, Alter G, Barouch DH. 2021. Correlates of protection against SARS-CoV-2 in rhesus macaques. Nature 590:630–634. 10.1038/s41586-020-03041-6. 33276369 PMC7906955

[10] Mercado NB, Zahn R, Wegmann F, Loos C, Chandrashekar A, Yu J, Liu J, Peter L, McMahan K, Tostanoski LH, He X, Martinez DR, Rutten L, Bos R, van Manen D, Vellinga J, Custers J, Langedijk JP, Kwaks T, Bakkers MJG, Zuijdgeest D, Rosendahl Huber SK, Atyeo C, Fischinger S, Burke JS, Feldman J, Hauser BM, Caradonna TM, Bondzie EA, Dagotto G, Gebre MS, Hoffman E, Jacob-Dolan C, Kirilova M, Li Z, Lin Z, Mahrokhian SH, Maxfield LF, Nampanya F, Nityanandam R, Nkolola JP, Patel S, Ventura JD, Verrington K, Wan H, Pessaint L, Van Ry A, Blade K, Strasbaugh A, Cabus M, et al. 2020. Single-shot Ad26 vaccine protects against SARS-CoV-2 in rhesus macaques. Nature 586:583–588. 10.1038/s41586-020-2607-z. 32731257 PMC7581548

[11] Korber B, Fischer WM, Gnanakaran S, Yoon H, Theiler J, Abfalterer W, Hengartner N, Giorgi EE, Bhattacharya T, Foley B, Hastie KM, Parker MD, Partridge DG, Evans CM, Freeman TM, de Silva TI, Sheffield C-GG, McDanal C, Perez LG, Tang H, Moon-Walker A, Whelan SP, LaBranche CC, Saphire EO, Montefiori DC, Sheffield COVID-19 Genomics Group. 2020. Tracking changes in SARS-CoV-2 spike: evidence that D614G increases infectivity of the COVID-19 virus. Cell 182:812–827.e19. 10.1016/j.cell.2020.06.043. 32697968 PMC7332439

[12] Zhou B, Thao TTN, Hoffmann D, Taddeo A, Ebert N, Labroussaa F, Pohlmann A, King J, Steiner S, Kelly JN, Portmann J, Halwe NJ, Ulrich L, Trueb BS, Fan X, Hoffmann B, Wang L, Thomann L, Lin X, Stalder H, Pozzi B, de Brot S, Jiang N, Cui D, Hossain J, Wilson MM, Keller MW, Stark TJ, Barnes JR, Dijkman R, Jores J, Benarafa C, Wentworth DE, Thiel V, Beer M. 2021. SARS-CoV-2 spike D614G change enhances replication and transmission. Nature 592:122–127. 10.1038/s41586-021-03361-1. 33636719

[13] Plante JA, Liu Y, Liu J, Xia H, Johnson BA, Lokugamage KG, Zhang X, Muruato AE, Zou J, Fontes-Garfias CR, Mirchandani D, Scharton D, Bilello JP, Ku Z, An Z, Kalveram B, Freiberg AN, Menachery VD, Xie X, Plante KS, Weaver SC, Shi PY. 2021. Spike mutation D614G alters SARS-CoV-2 fitness. Nature 592:116–121. 10.1038/s41586-020-2895-3. 33106671 PMC8158177

[14] Weissman D, Alameh M-G, de Silva T, Collini P, Hornsby H, Brown R, LaBranche CC, Edwards RJ, Sutherland L, Santra S, Mansouri K, Gobeil S, McDanal C, Pardi N, Hengartner N, Lin PJC, Tam Y, Shaw PA, Lewis MG, Boesler C, Şahin U, Acharya P, Haynes BF, Korber B, Montefiori DC. 2021. D614G spike mutation increases SARS CoV-2 susceptibility to neutralization. Cell Host Microbe 29:23–31.e4. 10.1016/j.chom.2020.11.012. 33306985 PMC7707640

[15] Kupferschmidt K. 2021. Fast-spreading U.K. virus variant raises alarms. Science 371:9–10. 10.1126/science.371.6524.9. 33384355

[16] Liu H, Zhang Q, Wei P, Chen Z, Aviszus K, Yang J, Downing W, Peterson S, Jiang C, Liang B, Reynoso L, Downey GP, Frankel SK, Kappler J, Marrack P, Zhang G. 2021. The basis of a more contagious 501Y.V1 variant of SARS-COV-2. bioRxiv 10.1101/2021.02.02.428884. PMC8063779 33893398

[17] Kemp SA, Collier DA, Datir R, Ferreira I, Gayed S, Jahun A, Hosmillo M, Rees-Spear C, Mlcochova P, Lumb IU, Roberts DJ, Chandra A, Temperton N, Sharrocks K, Blane E, Briggs J, van GM, Smith K, Bradley JR, Smith C, Doffinger R, Ceron-Gutierrez L, Barcenas-Morales G, Pollock DD, Goldstein RA, Smielewska A, Skittrall JP, Gouliouris T, Goodfellow IG, Gkrania-Klotsas E, Illingworth C, McCoy LE, Gupta RK. 2021. Neutralising antibodies in spike mediated SARS-CoV-2 adaptation. Nature 592:277–282. 10.1038/s41586-021-03291-y. 33545711 PMC7610568

[18] Kidd M, Richter A, Best A, Cumley N, Mirza J, Percival B, Mayhew M, Megram O, Ashford F, White T, Moles-Garcia E, Crawford L, Bosworth A, Atabani SF, Plant T, McNally A. 2021. S-variant SARS-CoV-2 lineage B1.1.7 is associated with significantly higher viral loads in samples tested by ThermoFisher TaqPath RT-qPCR. J Infect Dis 10.1093/infdis/jiab082. PMC7928763 33580259

[19] Liu Z, VanBlargan LA, Bloyet LM, Rothlauf PW, Chen RE, Stumpf S, Zhao H, Errico JM, Theel ES, Liebeskind MJ, Alford B, Buchser WJ, Ellebedy AH, Fremont DH, Diamond MS, Whelan SPJ. 2021. Identification of SARS-CoV-2 spike mutations that attenuate monoclonal and serum antibody neutralization. Cell Host Microbe 29:477–488.e4. 10.1016/j.chom.2021.01.014. 33535027 PMC7839837

[20] Yuan M, Huang D, Lee CD, Wu NC, Jackson AM, Zhu X, Liu H, Peng L, van Gils MJ, Sanders RW, Burton DR, Reincke SM, Pruss H, Kreye J, Nemazee D, Ward AB, Wilson IA. 2021. Structural and functional ramifications of antigenic drift in recent SARS-CoV-2 variants. bioRxiv 10.1101/2021.02.16.430500. PMC8284396 34016740

[21] Wang P, Nair MS, Liu L, Iketani S, Luo Y, Guo Y, Wang M, Yu J, Zhang B, Kwong PD, Graham BS, Mascola JR, Chang JY, Yin MT, Sobieszczyk M, Kyratsous CA, Shapiro L, Sheng Z, Huang Y, Ho DD. 2021. Increased resistance of SARS-CoV-2 variants B.1.351 and B.1.1.7 to antibody neutralization. bioRxiv 10.1101/2021.01.25.428137. 33684923

[22] Wu K, Werner AP, Koch M, Choi A, Narayanan E, Stewart-Jones GBE, Colpitts T, Bennett H, Boyoglu-Barnum S, Shi W, Moliva JI, Sullivan NJ, Graham BS, Carfi A, Corbett KS, Seder RA, Edwards DK. 2021. Serum neutralizing activity elicited by mRNA-1273 vaccine. N Engl J Med 384:1468–1470. 10.1056/NEJMc2102179. 33730471 PMC8008744

[23] Muik A, Wallisch A-K, Sänger B, Swanson KA, Mühl J, Chen W, Cai H, Maurus D, Sarkar R, Türeci Ö, Dormitzer PR, Şahin U. 2021. Neutralization of SARS-CoV-2 lineage B.1.1.7 pseudovirus by BNT162b2 vaccine-elicited human sera. Science 371:1152–1153. 10.1126/science.abg6105. 33514629 PMC7971771

[24] Liu Y, Liu J, Xia H, Zhang X, Fontes-Garfias CR, Swanson KA, Cai H, Sarkar R, Chen W, Cutler M, Cooper D, Weaver SC, Muik A, Sahin U, Jansen KU, Xie X, Dormitzer PR, Shi PY. 2021. Neutralizing activity of BNT162b2-elicited serum. N Engl J Med 384:1466–1468. 10.1056/NEJMc2102017. 33684280 PMC7944950

[25] Tada T, Dcosta BM, Zhou H, Vaill A, Kazmierski W, Landau NR. 2021. Decreased neutralization of SARS-CoV-2 global variants by therapeutic anti-spike protein monoclonal antibodies. bioRxiv 10.1101/2021.02.18.431897.

[26] Diamond M, Chen R, Xie X, Case J, Zhang X, VanBlargan L, Liu Y, Liu J, Errico J, Winkler E, Suryadevara N, Tahan S, Turner J, Kim W, Schmitz A, Thapa M, Wang D, Boon A, Pinto D, Presti R, O'Halloran J, Kim A, Deepak P, Fremont D, Corti D, Virgin H, Crowe J, Droit L, Ellebedy A, Shi PY, Gilchuk P. 2021. SARS-CoV-2 variants show resistance to neutralization by many monoclonal and serum-derived polyclonal antibodies. Res Sq 10.21203/rs.3.rs-228079/v1. PMC8058618 33664494

[27] Edara VV, Norwood C, Floyd K, Lai L, Davis-Gardner ME, Hudson WH, Mantus G, Nyhoff LE, Adelman MW, Fineman R, Patel S, Byram R, Gomes DN, Michael G, Abdullahi H, Beydoun N, Panganiban B, McNair N, Hellmeister K, Pitts J, Winters J, Kleinhenz J, Usher J, O'Keefe JB, Piantadosi A, Waggoner JJ, Babiker A, Stephens DS, Anderson EJ, Edupuganti S, Rouphael N, Ahmed R, Wrammert J, Suthar MS. 2021. Infection- and vaccine-induced antibody binding and neutralization of the B.1.351 SARS-CoV-2 variant. Cell Host Microbe 29:516–521.e3. 10.1016/j.chom.2021.03.009. 33798491 PMC7980225

[28] Piccoli L, Park YJ, Tortorici MA, Czudnochowski N, Walls AC, Beltramello M, Silacci-Fregni C, Pinto D, Rosen LE, Bowen JE, Acton OJ, Jaconi S, Guarino B, Minola A, Zatta F, Sprugasci N, Bassi J, Peter A, De Marco A, Nix JC, Mele F, Jovic S, Rodriguez BF, Gupta SV, Jin F, Piumatti G, Lo Presti G, Pellanda AF, Biggiogero M, Tarkowski M, Pizzuto MS, Cameroni E, Havenar-Daughton C, Smithey M, Hong D, Lepori V, Albanese E, Ceschi A, Bernasconi E, Elzi L, Ferrari P, Garzoni C, Riva A, Snell G, Sallusto F, Fink K, Virgin HW, Lanzavecchia A, Corti D, Veesler D. 2020. Mapping neutralizing and immunodominant sites on the SARS-CoV-2 spike receptor-binding domain by structure-guided high-resolution serology. Cell 183:1024–1042.e21. 10.1016/j.cell.2020.09.037. 32991844 PMC7494283

[29] Wu F, Liu M, Wang A, Lu L, Wang Q, Gu C, Chen J, Wu Y, Xia S, Ling Y, Zhang Y, Xun J, Zhang R, Xie Y, Jiang S, Zhu T, Lu H, Wen Y, Huang J. 2020. Evaluating the association of clinical characteristics with neutralizing antibody levels in patients who have recovered from mild COVID-19 in Shanghai, China. JAMA Intern Med 180:1356–1362. 10.1001/jamainternmed.2020.4616. 32808970 PMC9377417

[30] Wajnberg A, Amanat F, Firpo A, Altman DR, Bailey MJ, Mansour M, McMahon M, Meade P, Mendu DR, Muellers K, Stadlbauer D, Stone K, Strohmeier S, Simon V, Aberg J, Reich DL, Krammer F, Cordon-Cardo C. 2020. Robust neutralizing antibodies to SARS-CoV-2 infection persist for months. Science 370:1227–1230. 10.1126/science.abd7728. 33115920 PMC7810037

[31] Grifoni A, Weiskopf D, Ramirez SI, Mateus J, Dan JM, Moderbacher CR, Rawlings SA, Sutherland A, Premkumar L, Jadi RS, Marrama D, de Silva AM, Frazier A, Carlin AF, Greenbaum JA, Peters B, Krammer F, Smith DM, Crotty S, Sette A. 2020. Targets of T cell responses to SARS-CoV-2 coronavirus in humans with COVID-19 disease and unexposed individuals. Cell 181:1489–1501.e15. 10.1016/j.cell.2020.05.015. 32473127 PMC7237901

[32] Peng Y, Mentzer AJ, Liu G, Yao X, Yin Z, Dong D, Dejnirattisai W, Rostron T, Supasa P, Liu C, López-Camacho C, Slon-Campos J, Zhao Y, Stuart DI, Paesen GC, Grimes JM, Antson AA, Bayfield OW, Hawkins DEDP, Ker D-S, Wang B, Turtle L, Subramaniam K, Thomson P, Zhang P, Dold C, Ratcliff J, Simmonds P, de Silva T, Sopp P, Wellington D, Rajapaksa U, Chen Y-L, Salio M, Napolitani G, Paes W, Borrow P, Kessler BM, Fry JW, Schwabe NF, Semple MG, Baillie JK, Moore SC, Openshaw PJM, Ansari MA, Dunachie S, Barnes E, Frater J, Kerr G, Goulder P, ISARIC4C Investigators, et al. 2020. Broad and strong memory CD4(+) and CD8(+) T cells induced by SARS-CoV-2 in UK convalescent individuals following COVID-19. Nat Immunol 21:1336–1345. 10.1038/s41590-020-0782-6. 32887977 PMC7611020

[33] Rydyznski Moderbacher C, Ramirez SI, Dan JM, Grifoni A, Hastie KM, Weiskopf D, Belanger S, Abbott RK, Kim C, Choi J, Kato Y, Crotty EG, Kim C, Rawlings SA, Mateus J, Tse LPV, Frazier A, Baric R, Peters B, Greenbaum J, Ollmann Saphire E, Smith DM, Sette A, Crotty S. 2020. Antigen-specific adaptive immunity to SARS-CoV-2 in acute COVID-19 and associations with age and disease severity. Cell 183:996–1012.e19. 10.1016/j.cell.2020.09.038. 33010815 PMC7494270

[34] Brouwer PJM, Caniels TG, van der Straten K, Snitselaar JL, Aldon Y, Bangaru S, Torres JL, Okba NMA, Claireaux M, Kerster G, Bentlage AEH, van Haaren MM, Guerra D, Burger JA, Schermer EE, Verheul KD, van der Velde N, van der Kooi A, van Schooten J, van Breemen MJ, Bijl TPL, Sliepen K, Aartse A, Derking R, Bontjer I, Kootstra NA, Wiersinga WJ, Vidarsson G, Haagmans BL, Ward AB, de Bree GJ, Sanders RW, van Gils MJ. 2020. Potent neutralizing antibodies from COVID-19 patients define multiple targets of vulnerability. Science 369:643–650. 10.1126/science.abc5902. 32540902 PMC7299281

[35] Chi X, Yan R, Zhang J, Zhang G, Zhang Y, Hao M, Zhang Z, Fan P, Dong Y, Yang Y, Chen Z, Guo Y, Zhang J, Li Y, Song X, Chen Y, Xia L, Fu L, Hou L, Xu J, Yu C, Li J, Zhou Q, Chen W. 2020. A neutralizing human antibody binds to the N-terminal domain of the spike protein of SARS-CoV-2. Science 369:650–655. 10.1126/science.abc6952. 32571838 PMC7319273

[36] Liu L, Wang P, Nair MS, Yu J, Rapp M, Wang Q, Luo Y, Chan JF, Sahi V, Figueroa A, Guo XV, Cerutti G, Bimela J, Gorman J, Zhou T, Chen Z, Yuen KY, Kwong PD, Sodroski JG, Yin MT, Sheng Z, Huang Y, Shapiro L, Ho DD. 2020. Potent neutralizing antibodies against multiple epitopes on SARS-CoV-2 spike. Nature 584:450–456. 10.1038/s41586-020-2571-7. 32698192

[37] Klein SL, Pekosz A, Park HS, Ursin RL, Shapiro JR, Benner SE, Littlefield K, Kumar S, Naik HM, Betenbaugh MJ, Shrestha R, Wu AA, Hughes RM, Burgess I, Caturegli P, Laeyendecker O, Quinn TC, Sullivan D, Shoham S, Redd AD, Bloch EM, Casadevall A, Tobian AA. 2020. Sex, age, and hospitalization drive antibody responses in a COVID-19 convalescent plasma donor population. J Clin Invest 130:6141–6150. 10.1172/JCI142004. 32764200 PMC7598041

[38] Iyer AS, Jones FK, Nodoushani A, Kelly M, Becker M, Slater D, Mills R, Teng E, Kamruzzaman M, Garcia-Beltran WF, Astudillo M, Yang D, Miller TE, Oliver E, Fischinger S, Atyeo C, Iafrate AJ, Calderwood SB, Lauer SA, Yu J, Li Z, Feldman J, Hauser BM, Caradonna TM, Branda JA, Turbett SE, LaRocque RC, Mellon G, Barouch DH, Schmidt AG, Azman AS, Alter G, Ryan ET, Harris JB, Charles RC. 2020. Persistence and decay of human antibody responses to the receptor binding domain of SARS-CoV-2 spike protein in COVID-19 patients. Sci Immunol 5:eabe0367. 10.1126/sciimmunol.abe0367. 33033172 PMC7857394

[39] Seow J, Graham C, Merrick B, Acors S, Pickering S, Steel KJA, Hemmings O, O'Byrne A, Kouphou N, Galao RP, Betancor G, Wilson HD, Signell AW, Winstone H, Kerridge C, Huettner I, Jimenez-Guardeno JM, Lista MJ, Temperton N, Snell LB, Bisnauthsing K, Moore A, Green A, Martinez L, Stokes B, Honey J, Izquierdo-Barras A, Arbane G, Patel A, Tan MKI, O'Connell L, O'Hara G, MacMahon E, Douthwaite S, Nebbia G, Batra R, Martinez-Nunez R, Shankar-Hari M, Edgeworth JD, Neil SJD, Malim MH, Doores KJ. 2020. Longitudinal observation and decline of neutralizing antibody responses in the three months following SARS-CoV-2 infection in humans. Nat Microbiol 5:1598–1607. 10.1038/s41564-020-00813-8. 33106674 PMC7610833

[40] Chen Y, Zuiani A, Fischinger S, Mullur J, Atyeo C, Travers M, Lelis FJN, Pullen KM, Martin H, Tong P, Gautam A, Habibi S, Bensko J, Gakpo D, Feldman J, Hauser BM, Caradonna TM, Cai Y, Burke JS, Lin J, Lederer JA, Lam EC, Lavine CL, Seaman MS, Chen B, Schmidt AG, Balazs AB, Lauffenburger DA, Alter G, Wesemann DR. 2020. Quick COVID-19 healers sustain anti-SARS-CoV-2 antibody production. Cell 183:1496–1507.e16. 10.1016/j.cell.2020.10.051. 33171099 PMC7608032

[41] Roltgen K, Powell AE, Wirz OF, Stevens BA, Hogan CA, Najeeb J, Hunter M, Wang H, Sahoo MK, Huang C, Yamamoto F, Manohar M, Manalac J, Otrelo-Cardoso AR, Pham TD, Rustagi A, Rogers AJ, Shah NH, Blish CA, Cochran JR, Jardetzky TS, Zehnder JL, Wang TT, Narasimhan B, Gombar S, Tibshirani R, Nadeau KC, Kim PS, Pinsky BA, Boyd SD. 2020. Defining the features and duration of antibody responses to SARS-CoV-2 infection associated with disease severity and outcome. Sci Immunol 5:eabe0240. 10.1126/sciimmunol.abe0240. 33288645 PMC7857392

[42] Mateus J, Grifoni A, Tarke A, Sidney J, Ramirez SI, Dan JM, Burger ZC, Rawlings SA, Smith DM, Phillips E, Mallal S, Lammers M, Rubiro P, Quiambao L, Sutherland A, Yu ED, da Silva Antunes R, Greenbaum J, Frazier A, Markmann AJ, Premkumar L, de Silva A, Peters B, Crotty S, Sette A, Weiskopf D. 2020. Selective and cross-reactive SARS-CoV-2 T cell epitopes in unexposed humans. Science 370:89–94. 10.1126/science.abd3871. 32753554 PMC7574914

[43] Le Bert N, Tan AT, Kunasegaran K, Tham CYL, Hafezi M, Chia A, Chng MHY, Lin M, Tan N, Linster M, Chia WN, Chen MI, Wang LF, Ooi EE, Kalimuddin S, Tambyah PA, Low JG, Tan YJ, Bertoletti A. 2020. SARS-CoV-2-specific T cell immunity in cases of COVID-19 and SARS, and uninfected controls. Nature 584:457–462. 10.1038/s41586-020-2550-z. 32668444

